# Vitamin D Intake and Risk of Skin Cancer in US Women and Men

**DOI:** 10.1371/journal.pone.0160308

**Published:** 2016-08-24

**Authors:** Sang Min Park, Tricia Li, Shaowei Wu, Wen-Qing Li, Abrar A. Qureshi, Eunyoung Cho

**Affiliations:** 1 Channing Division of Network Medicine, Department of Medicine, Brigham and Women’s Hospital and Harvard Medical School, Boston, MA, United States of America; 2 Department of Family Medicine & Department of Biomedical Sciences, Seoul National University College of Medicine, Seoul, Korea; 3 Department of Dermatology, The Warren Alpert Medical School, Brown University, Providence, RI, United States of America; 4 Department of Epidemiology, Brown University School of Public Health, Providence, RI, United States of America; Indiana University Bloomington, UNITED STATES

## Abstract

Previous studies suggested a protective effect of vitamin D against skin cancer development. However, epidemiologic studies on orally taken vitamin D and risk of skin cancer (basal cell carcinoma [BCC], squamous cell carcinoma [SCC], and melanoma) are few. We prospectively evaluated whether total, dietary and supplemental vitamin D intake were associated with skin cancer risk based on 63,760 women in the Nurses' Health Study (1984–2010) and 41,530 men in the Health Professionals Follow-up Study (1986–2010). Dietary information on vitamin D intake was assessed every 2 to 4 years during the follow-up and cumulative averaged intake was used. We used Cox proportional hazard models to compute the hazard ratios (HR) and 95% confidence intervals (CI). Pooled HR of cohort-specific results were calculated using a random-effects model. During the follow-up, we documented 20,840 BCC, 2,329 SCC and 1,320 melanoma cases. Vitamin D consumption was not associated with the risk of SCC or melanoma but was modestly positively associated with BCC; the pooled HRs of BCC for extreme quintiles of vitamin D intake were 1.10 (95%CI = 1.05–1.15; *P*_trend_ = 0.05) for total vitamin D and 1.13 (95% CI = 1.07 to 1.20; *P*_trend_ <0.01) for dietary vitamin D. Stratified analysis according to sun exposure related factors showed similar results. In conclusion, vitamin D intake was positively associated with risk of BCC, while null associations were found with SCC and melanoma. Our data do not support a beneficial role of orally taken vitamin D on skin cancer carcinogenesis.

## Introduction

Vitamin D has been associated with decreased risk of several sites of cancers, including colorectal,[1] breast,[2, 3] and kidney cancers,[4] but its association with risk of skin cancer including melanoma and keratinocyte carcinoma (KC, formerly known as non-melanoma skin cancer), the most prevalent cancer in the United States (US)[5], is still unclear. Skin is one of the primary organ for vitamin D synthesis and metabolism.[6] Previous experimental studies have shown a protective effect of vitamin D against ultraviolet radiation–induced DNA damage and skin cancer development.[7, 8] Several researchers suggested that orally taken vitamin D could decrease the risk of skin cancer. [9, 10] However, epidemiologic studies on the association between vitamin D and skin cancer development have been limited and showed inconsistent results.[11–13] A recent meta-analysis on vitamin D intake and skin cancer found no association between higher vitamin D intake and melanoma and basal cell carcinoma (BCC).[14] To our knowledge no data exist on vitamin D intake and squamous cell carcinoma (SCC) risk. Most previous epidemiologic studies had focused on a single measurement of plasma 25(OH)D as a marker of vitamin D level[15–17], and a recent meta-analysis found a statistically significant positive association between risk of KC and serum 25(OH)D level.[14] As serum 25(OH)D levels may reflect the accumulative storage of bodily vitamin D obtained from different resources[18] and the main source of vitamin D is the exposure to sunlight (ultraviolet B; a risk factor for skin cancer) of the skin[19], the effect of ultraviolet radiation exposure may have confounded the associations[20–22].

In our study, we prospectively examined the association between vitamin D intake including intakes from both diet and supplements and risk of KC (including separate evaluation of SCC) and melanoma based on data from two cohort studies: the Nurses’ Health Study (NHS, 1984–2010) and the Health Professionals Follow-up Study (HPFS, 1986–2010).

## Methods

### Study Population

Details of the two cohorts have been described elsewhere.[11, 23, 24] In brief, the NHS was established in 1976, when 121,700 US female registered nurses, aged 30–55 years completed an initial questionnaire regarding their lifestyle and medical history. The HPFS was initiated in 1986 when 51,529 male health professionals aged 40–75 years completed a questionnaire that inquired about medical history and lifestyle practices in 1986. The questionnaire data was updated biennially in both cohorts, with the follow-up rates generally exceeding 90%. The present study was approved by the Institutional Review Boards of Brigham and Women’s Hospital and Harvard School of Public Health.

### Assessment of Vitamin D intake and other Dietary Consumption

To assess dietary intake, we used a food frequency questionnaire (FFQ) to collect dietary information in 1984 in the NHS, and every four years since 1986 in the NHS and HPFS. Participants were asked how often on average they had consumed each type of food during the previous year. Our nutrient database was primarily based on data from the US Department of Agriculture. In addition, information on current use and dose of multivitamins and specific supplements were collected biennially. Total vitamin D intake was calculated by adding together intakes from dietary vitamin D, vitamin D-specific supplements and multivitamins containing vitamin D. In validation studies, dietary intake assessed by using the FFQ has been demonstrated to be a valid estimator of food intake when compared with multiple diet records.[25, 26] The correlation coefficients ranged from 0.62–0.81 in the NHS and 0.58–0.88 in the HPFS between intakes of major dietary sources of vitamin D (skim milk, whole milk, fatty fish, and breakfast cereal) assessed on the FFQ and intakes assessed on two 1-week dietary records.[25, 26] Data on other dietary factors, including intakes of total energy, citrus, alcohol, and coffee consumption, was also collected by the FFQs.

### Assessment of Covariates

Biennially, we collected information on body weight, physical activity, and smoking history in both women and men and menopausal status and post-menopausal hormone use among women. Height information was collected in 1976 in NHS and in 1986 in HPFS. We also collected major skin cancer associated factors[21, 27], including family history of melanoma; natural hair color; number of moles; skin reaction to sun exposure as a child/adolescent; number of blistering sunburns; average time spent in direct sunlight since high school; and cumulative UV flux at residence since baseline.

### Assessment of Basal cell carcinoma (BCC), Squamous cell cancer (SCC), and Melanoma Cases

Biennially, participants had reported diagnoses of BCC, SCC, or melanoma during the previous 2 years. Participants who reported SCC or melanoma were asked for permission to review their medical and pathological reports. Study physicians reviewed their reports to confirm the diagnoses. As for self-reported cases of BCC, we could not obtain medical records, but previous validation studies in the two cohorts have demonstrated a high accuracy of self-reported BCC, with around 90% confirmed by histopathology records.[12, 28, 29]

### Statistical Analysis

We followed participants for incident BCC, SCC and melanoma starting from 1984 in the NHS and 1986 in the HPFS. We excluded participants who had missing data on vitamin D intake at baseline and 49,617 HPFS men and 81,685 NHS women served as the base population. Individuals with a history of any cancer were excluded. Due to small cases of KC and melanoma in non-white participants,[21] the analyses were restricted to Caucasians. After exclusions, 63,760 women and 41,530 men remained in the present study.

Person-years of follow-up were calculated from the return month of the baseline survey to the date of the first report of any cancer, date of death, or end of follow-up (June 1, 2010 for women; January 1, 2010 for men), whichever came first.

To better estimate long-term dietary intake and to minimize within-person variation, we used cumulative averages of vitamin D intake derived throughout the entire follow-up period. Energy-adjusted vitamin D intake calculated from regression-residual method was used to minimize the variation due to energy intake and its related measurement error.[30] Total and dietary vitamin D intakes were categorized into quintiles with the lowest quintile as a reference. Supplementary vitamin D intakes were categorized as following: none, 1–99, 100–199, 200–399, ≥ 400 IU/d.

Cox proportional hazard models were used to assess the hazard ratios (HR) and 95% confidence intervals (CI) of BCC, SCC and melanoma associated with total, dietary and supplementary vitamin D intake. In multivariate analyses of vitamin D intake, we adjusted for following possible confounders [31, 32] and other skin cancer risk factors [21, 27]: family history of melanoma (yes vs. no), natural hair color (red, blonde, light brown, dark brown, black), number of arm moles (0, 1–2, 3–9, ≥10), sunburn susceptibility as a child/adolescent (none/some redness, burn, painful burn/blisters), number of lifetime blistering sunburns (0, 1–4, 5–9, ≥10), average time spent in direct sunlight since high school (<2, 2–5, 6–9, ≥10 hrs/wk), cumulative UV flux since baseline (quintiles), body mass index (<25.0, 25.0–29.9, 30.0–34.9, ≥35.0 kg/m^2^), physical activity (quintiles), smoking status (never, past, current with 1–14, 15–24, or ≥25 cigarettes/d), intakes of total energy (quintiles), alcohol (0, 0.1–4.9, 5.0–9.9, 10.0–19.9, ≥20.0 g/d), coffee (0, <1, 1–2, ≥3 cup/d) and citrus intake (quintiles). Among women analyses were additionally adjusted for menopausal status and postmenopausal hormone use. Trend tests were conducted by assigning median values for each category and analyzing this value as a continuous variable in the models. We performed separately analyses for the HPFS and the NHS, and then calculated pooled HRs using a random-effects model. P values for heterogeneity were calculated using Q statistics.

We performed several sensitivity analyses. First, we calculated the HRs of KC or melanoma in association with consumption of major vitamin D-rich food items such as fish, dairy, milk (skimmed and whole), breakfast cereal and egg.[25, 26] Second, if the number of skin cancer cases is enough, we further categorized total and dietary vitamin D intakes into deciles. Second, we performed stratified analysis according to major sun exposure variables, including annual UV flux at residence, history of blistering sunburns, and average time spent in direct sunlight since high school. Statistical tests were performed using SAS software version 9.2 (SAS Institute Inc., Cary, North Carolina). All statistical tests were two-sided, and the significance level was set at P less than 0.05.

## Results

Age-adjusted characteristics of participants (63,760 women and 41,530 men) according to the vitamin D intake are shown in Table 1. Participants with higher intake of total vitamin D tended to be older and to have higher levels of physical activity and higher proportion of sunscreen use. Both men and women with lower total vitamin D intake were more likely to smoke, and consumed higher amount of alcohol intake.

**Table 1:** Baseline Characteristics of Study Participants According to Quintile of Total Vitamin D intake in the Nurses’ Health Study and Health Professionals Follow-Up Study.

	Quintile of Total Vitamin D intake				
	Q1	Q2	Q3	Q4	Q5
**Women (1984)**					
Number of participants	12,582	12,734	12,779	12,871	12,794
Age (year)	48.9(7.0)	49.5(7.1)	50.2(7.2)	50.3(7.2)	51.5(7.1)
Family history of melanoma, %	6.5	6.8	7.1	7.4	7.1
Red/blonde hair, %	15.5	15.6	15.4	15.3	16.6
Painful burn/blisters reaction as a child/adolescent, %	14.1	14.3	14.6	14.7	15.6
No. of blistering sunburns	8.5(7.0)	8.7(6.9)	8.7(6.9)	8.9(6.9)	8.8(7.0)
Use of sunscreen, %	20.2	22.2	23.9	25.8	28.3
Annual UV flux at residence (×10^−4^ RB count)	121.8(24.5)	121.6(24.2)	120.1(23.2)	121.4(24.1)	123.6(25.4)
Body mass index (kg/m^2^)	25.0(4.9)	25.2(4.8)	25.3(4.8)	25.0(4.7)	24.7(4.6)
Physical activity level (MET hrs/wk)	12.3(19.2)	12.7(18.2)	13.9(20.2)	14.5(19.4)	16.6(23.9)
Current smoking, %	30.1	24.5	22.8	22.1	21.1
Menopause status, %	46.2	45.8	45.8	46.2	46.8
Current hormones use in postmenopausal women,^a^ %	20.6	21.5	22.9	26.0	30.4
Total Vit D intake, IU/d	92.5(23.1)	159.0(17.3)	231.0(27.8)	382.0(62.8)	714.5(243.3)
Dietary Vit D intake, IU/d	92.6(23.7)	154.0(23.7)	216.3(42.4)	236.7(110.4)	235.7(124.3)
Supplemental Vit D intake, IU/d	0.5(5.2)	3.0(13.3)	13.3(43.2)	159.7(159.9)	455.6(223.3)
Total energy intake (kcal/d)	1644.1(539.9)	1787.8(513.5)	1806.1(544.5)	1896.6(515.3)	1611.7(473.1)
Alcohol intake (g/d)	8.7(13.7)	7.4(11.7)	6.3(10.4)	6.8(11.7)	6.3(10.2)
**Men (1986)**					
Number of participants	8,462	8,353	8,339	8,295	8,081
Age (year)	51.5(9.2)	52.6(9.5)	53.4(9.7)	53.8(9.7)	55.5(9.6)
Family history of melanoma, %	3.5	3.9	4.2	4.2	4.3
Red/blonde hair, %	12.9	12.8	13.9	13.7	13.3
Painful burn/blisters reaction as a child/adolescent, %	23.7	22.6	23.8	24.6	23.2
No. of blistering sunburns	12.8(12.1)	12.6(12.1)	13.0(12.0)	13.1(12.1)	12.7(12.1)
Annual UV flux at residence (×10^−4^ RB count)	131.0(27.2)	128.7(26.5)	128.1(26.4)	128.4(26.9)	129.3(27.3)
Body mass index (kg/m^2^)	25.2(5.2)	25.2(5.0)	25.1(4.9)	24.8(5.0)	24.7(5.0)
Physical activity level (MET hrs/wk)	18.6(30.7)	20.2(26.9)	21.3(31.3)	21.3(28.1)	24.0(32.1)
Current smoking, %	11.5	9.6	9.2	9.2	8.4
Total Vit D intake, IU/d	123.7(36.0)	214.2(22.7)	309.6(34.6)	489.0(70.0)	907.8(315.4)
Dietary Vit D intake, IU/d	122.6(36.9)	209.3(32.6)	289.2(62.3)	317.5(148.5)	369.9(217.0)
Supplemental Vit D intake, IU/d	2.7(12.1)	7.1(21.5)	23.7(59.7)	174.9(171.3)	493.1(311.0)
Total energy intake (kcal/d)	1,929.1(617.6)	1,997.9(619.0)	2,038.7(627.7)	2,126.1(616.7)	1,855.5(573.4)
Alcohol intake (g/d)	14.0(19.0)	12.2(16.0)	10.9(14.8)	11.6(16.1)	10.0(13.5)

NOTE: Except for data on age and number of participants, all data are standardized to the age distribution of the study population.

During 24–26 years of follow-up, we documented 20,840 incident BCCs 2,329 incident SCCs and 1,320 melanomas. The medians of total vitamin D intake for extreme quintiles were 124.8 and 638.2 IU/d for women and 156.0 and 775.3 IU/d for men, respectively. Contrary to hypothesis, total vitamin D intake was significantly associated with increased risk of BCC, even after multivariable adjustment (Table 2). The HRs of BCC for the extreme quintiles were 1.12 (95% CI = 1.05 to 1.19; *P*_trend_<0.01) in women and 1.07 (95% CI = 1.00 to 1.15; *P*_trend_ = 0.14) in men; the pooled HR was 1.10 (95% CI = 1.05 to 1.15; *P*_trend_ = 0.05). The multivariable pooled HRs of BCC for top versus bottom deciles were 1.14 (95% CI = 1.07 to 1.22; *P*_trend_ <0.01) for total vitamin D intake and 1.16 (95% CI = 1.09 to 1.24; *P*_trend_ <0.001) for dietary vitamin D intake. Total vitamin D intake was also positively associated with risk of SCC and melanoma in age-adjusted analysis. However, after adjustment for other risk factors, total vitamin D consumption was not significantly associated with the risk of SCC or melanoma.

**Table 2:** Hazard ratios (and 95% confidence intervals) of keratinocyte carcinoma and melanoma by total vitamin D intake in the Nurses’ Health Study (NHS), and Health Professionals Follow-Up Study (HPFS).

	Quintile of total vitamin D intake					P for trend
	1	2	3	4	5	
**Basal cell carcinoma**						
** NHS**						
** **Median total vitamin D, IU/d	124.8	210.9	304.5	437.4	638.2	
** **No. of cases	1862	2245	2530	2677	2493	
** **Person-years	249295	267002	268627	262065	234592	
** **Age-adjusted	1 (referent)	1.08 (1.01, 1.14)	1.18 (1.11, 1.25)	1.26 (1.18, 1.33)	1.27 (1.19, 1.34)	<0.0001
** **Multivariate-adjusted *	1 (referent)	1.02 (0.95, 1.08)	1.08 (1.02, 1.15)	1.13 (1.06, 1.20)	1.12 (1.05, 1.19)	<0.0001
** HPFS**						
** **Median total vitamin D, IU/d	156.0	253.5	363.2	519.9	775.3	
** **No. of cases	1552	1792	1926	1915	1848	
** **Person-years	146434	143684	141854	137579	127496	
** **Age-adjusted	1 (referent)	1.13 (1.05, 1.20)	1.19 (1.12, 1.28)	1.19 (1.12, 1.28)	1.18 (1.11, 1.27)	<0.0001
** **Multivariate-adjusted *	1 (referent)	1.05 (0.98, 1.13)	1.08 (1.01, 1.16)	1.06 (0.99, 1.14)	1.07 (1.00, 1.15)	0.14
** Pooled**^**†**^						
** **Age-adjusted	1 (referent)	1.10 (1.05, 1.15)	1.19 (1.13, 1.24)	1.23 (1.17, 1.29)	1.23 (1.15, 1.31)	<0.001
** **Multivariate-adjusted *	1 (referent)	1.03 (0.99, 1.08)	1.08 (1.04, 1.13)	1.10 (1.04, 1.16)	1.10 (1.05, 1.15)	0.05
**Squamous cell cancer**						
** NHS**						
** **No. of cases	198	254	273	222	245	
** **Person-years	250275	268142	269897	263488	235873	
** **Age-adjusted	1 (referent)	1.13 (0.94, 1.36)	1.18 (0.98, 1.41)	0.96 (0.79, 1.17)	1.18 (0.97, 1.42)	0.47
** **Multivariate-adjusted *	1 (referent)	1.04 (0.86, 1.25)	1.04 (0.87, 1.26)	0.83 (0.68, 1.01)	1.00 (0.82, 1.21)	0.33
** HPFS**						
** **No. of cases	195	234	264	210	234	
** **Person-years	147775	145240	143482	139252	129070	
** **Age-adjusted	1 (referent)	1.14 (0.94, 1.38)	1.27 (1.05, 1.53)	0.99 (0.82, 1.21)	1.14 (0.94, 1.38)	0.71
** **Multivariate-adjusted *	1 (referent)	1.04 (0.86, 1.25)	1.11 (0.92, 1.34)	0.85 (0.70, 1.04)	1.04 (0.85, 1.26)	0.61
** Pooled**^**†**^						
** **Age-adjusted	1 (referent)	1.13 (0.99, 1.29)	1.22 (1.07, 1.39)	0.98 (0.85, 1.12)	1.16 (1.01, 1.33)	0.45
** **Multivariate-adjusted *	1 (referent)	1.04 (0.91, 1.19)	1.07 (0.94, 1.23)	0.84 (0.73, 0.96)	1.02 (0.89, 1.17)	0.30
**Melanoma**						
** NHS**						
** **No. of cases	114	170	158	153	144	
** **Person-years	250335	268208	269995	263538	235957	
** **Age-adjusted	1 (referent)	1.36 (1.07, 1.73)	1.25 (0.98, 1.59)	1.25 (0.98, 1.59)	1.33 (1.04, 1.71)	0.14
** **Multivariate-adjusted *	1 (referent)	1.24 (0.98, 1.58)	1.10 (0.86, 1.40)	1.09 (0.85, 1.39)	1.15 (0.90, 1.49)	0.74
** HPFS**						
** **No. of cases	105	115	122	122	117	
** **Person-years	147859	145335	143613	139301	129155	
** **Age-adjusted	1 (referent)	1.09 (0.84, 1.42)	1.16 (0.89, 1.50)	1.17 (0.90, 1.52)	1.19 (0.91, 1.55)	0.21
** **Multivariate-adjusted *	1 (referent)	0.99 (0.76, 1.30)	1.01 (0.77, 1.31)	0.99 (0.76, 1.29)	1.03 (0.78, 1.35)	0.85
** Pooled**^**†**^						
** **Age-adjusted	1 (referent)	1.23 (0.99, 1.52)	1.21 (1.01, 1.44)	1.21 (1.01, 1.45)	1.27 (1.06, 1.52)	0.05
** **Multivariate-adjusted *	1 (referent)	1.12 (0.90, 1.39)	1.05 (0.88, 1.26)	1.04 (0.87, 1.24)	1.09 (0.91, 1.31)	0.71

* Adjusted for family history of melanoma, natural hair color, number of arm moles, sunburn susceptibility as a child/adolescent, number of lifetime blistering sunburns, average time spent in direct sunlight since high school, cumulative UV flux since baseline, body mass index, physical activity, smoking status, intakes of total energy, alcohol, coffee and citrus intake. Among women analyses were additionally adjusted for menopausal status and postmenopausal hormone use.

^**†**^ The multivariate-adjusted hazard ratios from each cohort were pooled using random effects model.

Higher intakes of vitamin D from food and from supplements were also each associated with increased risk of BCC. The multivariable pooled HR of BCC for top versus bottom quintiles of dietary vitamin D intake was 1.13 (95% CI = 1.07 to 1.20; *P*_trend_ <0.01) in a dose-dependent manner (Table 3). Participants who took supplementary vitamin D ≥ 400 IU/d also had a higher risk of BCC (pooled HR = 1.07, 95% CI = 1.03 to 1.12; *P*_trend_ = 0.03) than non-users (Table 4). In a sensitivity analysis adjusting for both dietary vitamin D intake and supplemental vitamin D intake in the model simultaneously, no substantial differences in trends were found. In multivariate analysis, dietary or supplemental vitamin D consumption did not show any protective effects on SCC or melanoma incidence (Tables 3 and 4), while a non-significant increased risk was found with higher intake of vitamin D from food for SCC (pooled HR = 1.14, 95% CI = 0.95 to 1.36; *P*_trend_ = 0.41) and melanoma (pooled HR = 1.06, 95% CI = 0.88 to 1.28; *P*_trend_ = 0.55).

**Table 3:** Hazard ratios (and 95% confidence intervals) of skin cancer by dietary vitamin D intake in the Nurses’ Health Study (NHS), and Health Professionals Follow-Up Study (HPFS).

	Quintile of dietary vitamin D intake					P for trend
	1	2	3	4	5	
**Basal cell carcinoma**						
** NHS**						
** **Median dietary vitamin D, IU/d	97.6	145.0	185.2	233.6	314.4	
** **No. of cases	1963	2274	2583	2542	2445	
** **Person-years	253052	264672	265341	259109	239408	
** **Age-adjusted	1 (referent)	1.07 (1.01, 1.14)	1.18 (1.11, 1.25)	1.16 (1.09, 1.23)	1.18 (1.11, 1.25)	<0.0001
** **Multivariate-adjusted *	1 (referent)	1.03 (0.97, 1.10)	1.13 (1.06, 1.20)	1.11 (1.04, 1.18)	1.13 (1.07, 1.20)	<0.001
** HPFS**						
** **Median dietary vitamin D, IU/d	126.3	192.3	245.8	311.0	433.8	
** **No. of cases	1619	1843	1829	1895	1847	
** **Person-years	148149	144004	139649	136953	128294	
** **Age-adjusted	1 (referent)	1.13 (1.05, 1.20)	1.13 (1.06, 1.21)	1.17 (1.10, 1.25)	1.16 (1.09, 1.25)	<0.0001
** **Multivariate-adjusted *	1 (referent)	1.09 (1.02, 1.16)	1.07 (1.00, 1.15)	1.10 (1.03, 1.18)	1.12 (1.04, 1.20)	<0.01
** Pooled**^**†**^						
** **Age-adjusted	1 (referent)	1.10 (1.04, 1.15)	1.16 (1.11, 1.21)	1.17 (1.12, 1.22)	1.17 (1.12, 1.23)	<0.0001
** **Multivariate-adjusted *	1 (referent)	1.06 (1.01, 1.11)	1.10 (1.05, 1.16)	1.10 (1.06, 1.15)	1.13 (1.08, 1.18)	<0.001
**Squamous cell cancer**						
** NHS**						
** **No. of cases	173	284	251	249	235	
** **Person-years	254082	265801	266686	260457	240649	
** **Age-adjusted	1 (referent)	1.51 (1.25, 1.82	1.29 (1.06, 1.56)	1.28 (1.06, 1.56)	1.29 (1.06, 1.57)	0.28
** **Multivariate-adjusted *	1 (referent)	1.45 (1.20, 1.76)	1.23 (1.01, 1.49)	1.23 (1.01, 1.50)	1.25 (1.02, 1.52)	0.43
** HPFS**						
** **No. of cases	222	237	213	231	234	
** **Person-years	149546	145599	141188	138588	129897	
** **Age-adjusted	1 (referent)	1.05 (0.87, 1.26)	0.94 (0.78, 1.13)	1.02 (0.85, 1.22)	1.06 (0.87, 1.27)	0.65
** **Multivariate-adjusted *	1 (referent)	1.00 (0.83, 1.21)	0.89 (0.73, 1.07)	0.97 (0.80, 1.17)	1.04 (0.86, 1.26)	0.66
** Pooled**^**†**^						
** **Age-adjusted	1 (referent)	1.26 (0.88, 1.79)	1.10 (0.81, 1.50)	1.14 (0.91, 1.43)	1.16 (0.95, 1.41)	0.31
** **Multivariate-adjusted *	1 (referent)	1.21 (0.84, 1.73)	1.04 (0.76, 1.43)	1.09 (0.86, 1.38)	1.14 (0.95, 1.36)	0.41
**Melanoma**						
** NHS**						
** **No. of cases	113	151	169	184	122	
** **Person-years	254140	265923	266738	260503	240729	
** **Age-adjusted	1 (referent)	1.27 (0.99, 1.61)	1.40 (1.11, 1.78)	1.56 (1.23, 1.97)	1.13 (0.87, 1.46)	0.25
** **Multivariate-adjusted *	1 (referent)	1.18 (0.93, 1.51)	1.29 (1.01, 1.64)	1.43 (1.12, 1.81)	1.05 (0.81, 1.36)	0.56
** HPFS**						
** **No. of cases	105	125	115	122	114	
** **Person-years	149652	145686	141264	138675	129987	
** **Age-adjusted	1 (referent)	1.19 (0.92, 1.55)	1.13 (0.87, 1.48)	1.22 (0.94, 1.58)	1.18 (0.90, 1.54)	0.29
** **Multivariate-adjusted *	1 (referent)	1.14 (0.87, 1.47)	1.04 (0.80, 1.36)	1.10 (0.84, 1.43)	1.08 (0.82, 1.41)	0.76
** Pooled**^**†**^						
** **Age-adjusted	1 (referent)	1.23 (1.03, 1.47)	1.27 (1.03, 1.57)	1.39 (1.09, 1.77)	1.15 (0.96, 1.39)	0.12
** **Multivariate-adjusted *	1 (referent)	1.16 (0.97, 1.39)	1.17 (0.95, 1.43)	1.26 (0.98, 1.62)	1.06 (0.88, 1.28)	0.55

* Adjusted for family history of melanoma, natural hair color, number of arm moles, sunburn susceptibility as a child/adolescent, number of lifetime blistering sunburns, average time spent in direct sunlight since high school, cumulative UV flux since baseline, body mass index, physical activity, smoking status, intakes of total energy, alcohol, coffee and citrus intake. Among women analyses were additionally adjusted for menopausal status and postmenopausal hormone use.

^**†**^ The multivariate-adjusted hazard ratios from each cohort were pooled using random effects model.

**Table 4:** Hazard ratios (and 95% confidence intervals) of skin cancer by supplemental vitamin D intake in the Nurses’ Health Study (NHS), and Health Professionals Follow-Up Study (HPFS).

	Category of supplemental vitamin D intake					P for trend
	None	1–99 IU/d	100–199 IU/d	200–399 IU/d	≥ 400 IU/d	
**Basal cell carcinoma**						
** NHS**						
** **Median supplemental vitamin D, IU/d	0	57.1	133.3	266.2	400.0	
** **No. of cases	3461	1879	2046	2916	1505	
** **Person-years	473758	198597	205885	265126	138215	
** **Age-adjusted	1 (referent)	1.07 (1.00, 1.13)	1.12 (1.05, 1.18)	1.22 (1.16, 1.28)	1.26 (1.19, 1.34)	<0.0001
** **Multivariate-adjusted *	1 (referent)	1.02 (0.96, 1.08)	1.03 (0.97, 1.09)	1.07 (1.02, 1.13)	1.09 (1.03, 1.16)	<0.001
** HPFS**						
** **Median supplemental vitamin D, IU/d	0	56.0	133.3	251.2	400.0	
** **No. of cases	3584	1243	1221	1712	1273	
** **Person-years	321957	91928	79240	116545	87379	
** **Age-adjusted	1 (referent)	1.09 (1.02, 1.17)	1.19 (1.11, 1.27)	1.14 (1.08, 1.21)	1.19 (1.12, 1.27)	<0.0001
** **Multivariate-adjusted *	1 (referent)	1.05 (0.99, 1.13)	1.10 (1.02, 1.17)	1.02 (0.96, 1.08)	1.05 (0.98, 1.12)	0.27
** Pooled**^**†**^						
** **Age-adjusted	1 (referent)	1.08 (1.03, 1.13)	1.15 (1.08, 1.22)	1.18 (1.11, 1.26)	1.23 (1.16, 1.30)	<0.0001
** **Multivariate-adjusted *	1 (referent)	1.03 (0.99, 1.08)	1.06 (0.99, 1.13)	1.05 (0.99, 1.10)	1.07 (1.03, 1.12)	0.03
**Squamous cell cancer**						
** NHS**						
** **No. of cases	264	238	232	319	139	
** **Person-years	475608	199572	206913	266604	138978	
** **Age-adjusted	1 (referent)	1.14 (0.95, 1.38)	1.13 (0.94, 1.36)	1.22 (1.03, 1.45)	1.31 (1.06, 1.61)	0.01
** **Multivariate-adjusted *	1 (referent)	1.04 (0.86, 1.26)	0.96 (0.79, 1.16)	0.98 (0.82, 1.17)	1.00 (0.80, 1.23)	0.70
** HPFS**						
** **No. of cases	427	160	165	235	150	
** **Person-years	325089	92992	80290	117993	88455	
** **Age-adjusted	1 (referent)	0.96 (0.80, 1.16)	1.05 (0.87, 1.26)	1.07 (0.91, 1.26)	1.13 (0.93, 1.36)	0.13
** **Multivariate-adjusted *	1 (referent)	0.91 (0.76, 1.10)	0.92 (0.76, 1.11)	0.88 (0.75, 1.04)	0.92 (0.76, 1.11)	0.24
** Pooled**^**†**^						
** **Age-adjusted	1 (referent)	1.05 (0.88, 1.24)	1.09 (0.95, 1.24)	1.14 (1.01, 1.29)	1.20 (1.04, 1.40)	<0.01
** **Multivariate-adjusted *	1 (referent)	0.98 (0.85, 1.11)	0.94 (0.82, 1.07)	0.92 (0.82, 1.04)	0.95 (0.82, 1.09)	0.26
**Melanoma**						
** NHS**						
** **No. of cases	204	140	130	176	89	
** **Person-years	475624	199644	207017	266724	139024	
** **Age-adjusted	1 (referent)	1.04 (0.83, 1.31)	1.01 (0.80, 1.27)	1.09 (0.88, 1.35)	1.28 (1.00, 1.65)	0.07
** **Multivariate-adjusted *	1 (referent)	0.97 (0.77, 1.22)	0.89 (0.70, 1.12)	0.93 (0.74, 1.15)	1.08 (0.83, 1.40)	0.83
** HPFS**						
** **No. of cases	221	94	77	113	76	
** **Person-years	325265	93044	80356	118100	88498	
** **Age-adjusted	1 (referent)	1.21 (0.94, 1.55)	1.09 (0.83, 1.42)	1.16 (0.92, 1.46)	1.20 (0.92, 1.55)	0.18
** **Multivariate-adjusted *	1 (referent)	1.15 (0.89, 1.48)	0.97 (0.74, 1.28)	0.98 (0.77, 1.24)	1.01 (0.78, 1.32)	0.78
** Pooled**^**†**^						
** **Age-adjusted	1 (referent)	1.12 (0.94, 1.32)	1.04 (0.87, 1.24)	1.12 (0.96, 1.31)	1.24 (1.03, 1.49)	0.02
** **Multivariate-adjusted *	1 (referent)	1.05 (0.88, 1.24)	0.93 (0.78, 1.11)	0.95 (0.81, 1.12)	1.04 (0.87, 1.26)	0.97

^*^ Adjusted for family history of melanoma, natural hair color, number of arm moles, sunburn susceptibility as a child/adolescent, number of lifetime blistering sunburns, average time spent in direct sunlight since high school, cumulative UV flux since baseline, body mass index, physical activity, smoking status, intakes of total energy, alcohol, coffee and citrus intake. Among women analyses were additionally adjusted for menopausal status and postmenopausal hormone use.

^**†**^ The multivariate-adjusted hazard ratios from each cohort were pooled using random effects model.

Stratified analysis according to sun exposure related factors showed similar trends between vitamin D intake and skin cancers (S1 Table).

Among the major vitamin D-contributing food items, dietary intakes of total fish, low fat/skim milk, and cereal showed significant positive associations with risk of BCC (Table 5). The pooled multivariable HRs of BCC for top versus bottom quintiles was 1.11 (95% CI: 1.06 to 1.17; *P*_trend_<0.01) for fish intake, 1.12 (95% CI: 1.06 to 1.17; *P*_trend_<0.01) for low fat/skimmed milk intake, and 1.13 (95% CI: 1.01 to 1.27; *P*_trend_<0.01) for breakfast cereal intake, respectively. Neither total dairy food intake nor egg consumption were significantly associated with risk of BCC. Dietary intakes of vitamin D-rich food items were not significantly associated with the risk of SCC or melanoma in a dose-dependent manner (S2 Table). Marginal increased risks for melanoma were found with higher intake of total fish (pooled HR for top versus bottom quintiles = 1.17, 95% CI = 0.98 to 1.41; *P*_trend_ = 0.12), white fish (pooled HR = 1.27, 95% CI = 1.05 to 1.54; *P*_trend_ = 0.07) and breakfast cereal (pooled HR = 1.19, 95% CI = 0.98 to 1.45; *P*_trend_ = 0.10).

**Table 5:** Multivariate-adjusted Hazard ratios* (and 95% confidence intervals) of Basal Cell Carcinoma by intake of vitamin D rich food in the Nurses’ Health Study (NHS), and Health Professionals Follow-Up Study (HPFS).

	Quintile of vitamin D rich food intake					P for trend
	1	2	3	4	5	
**Total Fish**						
** NHS**	1 (referent)	1.07 (1.01, 1.13)	1.08 (1.02, 1.15)	1.12 (1.05, 1.19)	1.10 (1.03, 1.17)	*<0*.*01*
** HPFS**	1 (referent)	1.03 (0.96, 1.10)	1.02 (0.94, 1.09)	1.07 (0.99, 1.14)	1.14 (1.06, 1.23)	*<0*.*01*
** Pooled**^**†**^	1 (referent)	1.05 (1.00, 1.10)	1.06 (0.99, 1.13)	1.10 (1.04, 1.16)	1.12 (1.07, 1.17)	*<0*.*01*
**White Fish**						
** NHS**	1 (referent)	1.02 (0.95, 1.08)	1.01 (0.95, 1.07)	1.05 (0.99, 1.11)	1.04 (0.97, 1.11)	0.14
** HPFS**	1 (referent)	1.00 (0.94, 1.07)	1.10 (1.01, 1.20)	1.05 (0.98, 1.12)	1.07 (1.00, 1.15)	0.07
** Pooled**^**†**^	1 (referent)	1.01 (0.96, 1.06)	1.04 (0.97, 1.11)	1.05 (1.00, 1.09)	1.05 (1.00, 1.10)	*<0*.*01*
**Tuna**						
** NHS**	1 (referent)	1.03 (0.97, 1.09)	1.08 (1.02, 1.14)	1.08 (1.02, 1.14)	1.08 (1.01, 1.14)	*0*.*01*
** HPFS**	1 (referent)	0.99 (0.92, 1.05)	1.03 (0.95, 1.12)	1.08 (1.00, 1.15)	1.12 (1.04, 1.19)	*<0*.*01*
** Pooled**^**†**^	1 (referent)	1.01 (0.96, 1.06)	1.06 (1.02, 1.12)	1.08 (1.03, 1.13)	1.09 (1.05, 1.14)	*<0*.*01*
**Total Dairy Food**						
** NHS**	1 (referent)	0.99 (0.93, 1.05)	1.01 (0.95, 1.08)	1.01 (0.94, 1.07)	1.04 (0.97, 1.11)	0.27
** HPFS**	1 (referent)	1.04 (0.97, 1.11)	1.05 (0.98, 1.13)	1.02 (0.95, 1.10)	1.01 (0.93, 1.09)	0.97
** Pooled**^**†**^	1 (referent)	1.01 (0.96, 1.06)	1.03 (0.99, 1.08)	1.01 (0.97, 1.06)	1.02 (0.97, 1.08)	*0*.*41*
**Total Milk**						
** NHS**	1 (referent)	1.06 (1.00, 1.12)	1.07 (1.00, 1.13)	1.07 (1.01, 1.14)	1.09 (1.02, 1.16)	0.02
** HPFS**	1 (referent)	1.06 (0.99, 1.14)	1.04 (0.98, 1.11)	1.10 (1.03, 1.18)	1.00 (0.93, 1.07)	0.58
** Pooled**^**†**^	1 (referent)	1.06 (1.01, 1.11)	1.05 (1.01, 1.10)	1.08 (1.04, 1.13)	1.04 (0.96, 1.13)	*0*.*14*
**Skim or low fat Milk**						
** NHS**	1 (referent)	1.11 (1.04, 1.18)	1.11 (1.04, 1.18)	1.11 (1.04, 1.18)	1.14 (1.07, 1.22)	<0.01
** HPFS**	1 (referent)	1.16 (1.08, 1.24)	1.11 (1.03, 1.19)	1.16 (1.08, 1.24)	1.09 (1.01, 1.17)	0.06
** Pooled**^**†**^	1 (referent)	1.13 (1.08, 1.19)	1.11 (1.06, 1.16)	1.13 (1.08, 1.18)	1.12 (1.06, 1.17)	*<0*.*01*
**Breakfast Cereal**						
** NHS**	1 (referent)	1.01 (0.95, 1.07)	1.01 (0.94, 1.07)	1.09 (1.03, 1.16)	1.07 (1.01, 1.14)	*<0*.*01*
** HPFS**	1 (referent)	1.03 (0.96, 1.11)	1.11 (1.04, 1.19)	1.16 (1.08, 1.25)	1.20 (1.12, 1.29)	*<0*.*01*
** Pooled**^**†**^	1 (referent)	1.02 (0.97, 1.07)	1.06 (0.96, 1.17)	1.12 (1.06, 1.19)	1.13 (1.01, 1.27)	*<0*.*01*
**Egg**						
** NHS**	1 (referent)	0.98 (0.93, 1.04)	1.01 (0.95, 1.07)	0.98 (0.93, 1.04)	0.95 (0.88, 1.02)	0.15
** HPFS**	1 (referent)	0.96 (0.89, 1.02)	1.03 (0.96, 1.10)	1.02 (0.96, 1.09)	0.97 (0.89, 1.05)	0.66
** Pooled**^**†**^	1 (referent)	0.97 (0.93, 1.01)	1.02 (0.98, 1.06)	1.00 (0.96, 1.04)	0.96 (0.91, 1.01)	*0*.*60*

^*^ Adjusted for family history of melanoma, natural hair color, number of arm moles, sunburn susceptibility as a child/adolescent, number of lifetime blistering sunburns, average time spent in direct sunlight since high school, cumulative UV flux since baseline, body mass index, physical activity, smoking status, intakes of total energy, alcohol, coffee and citrus intake. Among women analyses were additionally adjusted for menopausal status and postmenopausal hormone use.

^**†**^ The multivariate-adjusted hazard ratios from each cohort were pooled using random effects model.

## Discussion

In these large population-based cohort studies, we found that orally taken vitamin D did not exert any protective effect against skin cancer development. Higher intakes of vitamin D from food and from supplements were associated with increased risk of BCC of the skin, while a non-significant increased risk was found with SCC and melanoma. These trends remained despite strategies to minimize confounding by sun exposure. When we examined individual vitamin D-rich foods, intakes of fish, cereal and skim milk were significantly associated with risk of BCC in both cohorts.

Most previous studies on vitamin D and risk of skin cancer used a single measurement of plasma 25(OH)D as a marker of vitamin D level. [9, 14, 16, 17, 33] In a recent meta-analysis, the summary relative risks for the association of highest versus lowest quintile of serum vitamin D levels was 1.64 (95% CI 1.02–2.65) for KC and 1.46 (95% CI 0.60–3.53) for melanoma.[14] However, one-time measurement of plasma vitamin D levels is known to mainly reflect sun exposure,[18] and may not reflect longer-term vitamin D status. Also, many of the studies in the meta-analysis lacked adjustment for sun exposure related factors and pigmentary traits. Thus, the positive association might be confounded by these factors, especially sun exposure related factors. While sun exposure is a known risk factor for skin cancer development, a potential protective effect of orally taken vitamin D against skin cancer development has been raised based on the evidence from experimental studies. Several animal studies have demonstrated that vitamin D treatment decreases cell growth and metastasis of skin cancer.[34, 35] Other studies have suggested that vitamin D may protect the skin against ultraviolet radiation–induced DNA damage.[7, 8][36] However, in our study, orally taken vitamin D from diet and supplements did not appear to exert any protective effect against KC or melanoma development. Vitamin D intake was rather positively associated with BCC risk. A meta-analysis of cohort/case-control studies and a randomized clinical trial of vitamin D intake and risks of melanoma and KC (largely BCC) found null associations of vitamin D intake with skin cancer.[14] The meta-analysis included 5 studies of melanoma and 4 studies of BCC and KC (without separate evaluation of BCC and SCC). The study included earlier follow-up of NHS and HPFS.[11, 12] The sample size of our study was much larger than the meta-analysis, especially for BCC. Thus, we had a better statistical power to detect an association. Furthermore, to our knowledge, our study was the first evaluating vitamin D intake in relation to SCC risk.

Our findings suggest that even intake of vitamin D from diet and/or supplements may be associated with increased risk of BCC. Earlier studies from our group that examined the association of risk of BCC and intakes of multiple nutrients in the NHS (4-years of follow-up)[11] and HPFS (8-years of follow-up)[12] did not find any significant association for intakes of vitamin D. However, the number of cases was much smaller in the previous investigations (female 771 cases; male 3190 cases) than the present updated analysis. A previous clinical trial did not find a protective effect of calcium/vitamin D supplements on risk of skin cancer either. In the Women's Health Initiative calcium/vitamin D clinical trial, daily supplementation with 1,000 mg of calcium and 400 IU of vitamin D for a mean follow-up period of 7.0 years had no effect on KC incidence.[37] Also, since the study evaluated calcium and vitamin D together at one dose, it was unknown whether vitamin D per se or vitamin D at different doses would be effective.

The discrepancy between our results and experimental data in animals might be hard to interpret. Besides orally consumed vitamin D, large amount of vitamin D would be synthesized by sunlight exposure.[19] However, because sunlight exposure also elevates the risk of skin cancer,[20–22] the beneficial effect of vitamin D might be canceled out. Based on our data, it appears that orally taken vitamin D per se is not associated with a reduced risk of skin cancer. In animal models of cutaneous carcinogenesis, high-dose vitamin D treatment in short time periods prior to irradiation with UV was used,[7] [36] while orally taken vitamin D in the epidemiologic studies may represent chronic low-dose exposure. These differences in the timing and the dose of the exposure may lead to different effects on skin cancer development, but little is known about these associations and further research will be needed.

Although we found a modest positive association between vitamin D intake and BCC risk, the major differences in BCC risk were largely found in lower vitamin D intake quintiles, while the risks were similar among higher quintiles of vitamin D intake. Therefore, the positive association was largely driven by the effect of low vitamin D intake. We cannot exclude the possibility of reporting bias and misclassification of BCC, because the identification of BCC cases in this study was based on self-report. Although previous studied have shown a high accuracy of self-reported BCC (high sensitivity of self-reporting) [12, 28] it is unclear whether self-reporting is highly specific as well. Our participants in the highest quintile of vitamin D intake were less likely to smoke and drink alcohol, which seemed that they were more health-conscious. It is possible that those in the highest quintile of vitamin D intake might be more likely to get skin examination and diagnosis of BCC, which could lead to a HR biased towards positive values. In addition, participants in the highest quintile of vitamin D intake were most likely to be physically active, which could be associated with getting more sun exposure.

On the other hand, other components in vitamin D-rich foods may be responsible for the positive association between vitamin D intake and BCC. Among vitamin D-rich food items, dietary consumptions of fish and cereal were significantly associated with risk of BCC in both cohorts. In the United States, fish and breakfast cereals including rice are important dietary sources of arsenic,[38, 39] which is a possible environmental risk factor of skin cancer by causing oxidative stress, impairing immune function and increasing genotoxicity.[40, 41] A cohort study in Europe suggested that workplace co-exposure to arsenic and sunlight might be associated with an increased risk of KC.[42] Another case-control study also showed a positive association between BCC and arsenic exposure in drinking water.[43] However, there are few epidemiologic studies to assess the association between dietary arsenic exposure and skin cancer. We also found that intake of low fat/skim milk was associated with increased risk of BCC, while total dairy intake did not show any association. These findings disagree with a previous study that showed a positive association between consumption of unmodified dairy foods with high fat and KC risk.[44] Although higher milk intake has been associated with risk of cancer of several organs including liver [45], ovary[46] and prostate[47], little is known about the underlying mechanism of the potential milk-KC association. Further studies are needed to replicate this association in other populations and to explore the underlying mechanisms.

Our study had several limitations. First, our study population consisted of whites, well-educated health professionals, which may limit the generalizability of our results. Second, although we controlled for several strong predictors of skin cancer risk including the cumulative UV flux at residence, average time spent in direct sunlight since high school, number of moles on arms, skin reaction to sun exposure, and number of blistering sunburns, we cannot exclude residual confounding by sun-exposure, which is the primary risk factor for KC and melanoma and difficult to measure accurately. Third, the case numbers of SCC and melanoma were not as high as that of BCC, and we could not exclude the possibility of limited statistical power for those cancers.

In conclusion, we found no evidence that orally taken vitamin D plays an important protective role against the incidence of KC and cutaneous melanoma. Vitamin D intake was significantly and positively associated with risk of BCC. Further research is warranted to confirm our findings and identify the potential mechanisms underlying these associations.

## Supporting Information

S1 Table Pooled hazard ratios* (and 95% confidence intervals) of skin cancer by vitamin D intake stratified by sun exposure related factors in the Nurses’ Health Study (NHS), and Health Professionals Follow-Up Study (HPFS). (DOC)

S2 Table Pooled hazard ratios* (and 95% confidence intervals) of Squamous Cell Carcinoma and Melanoma by intake of vitamin D rich food in the Nurses’ Health Study (NHS), and Health Professionals Follow-Up Study (HPFS). (DOC)
