# Supplementary material for: Vitamin D Intake and Risk of Skin Cancer in US Women and Men
Source: PLoS One. 2016 Aug 24;11(8):e0160308. doi: 10.1371/journal.pone.0160308 (PMC4996491; doi:10.1371/journal.pone.0160308)
Supplement: S1 Table — (DOC) [file pone.0160308.s001.doc]

**Supplemental Table 1. Pooled hazard ratios^*^ (and 95% confidence intervals) of skin cancer by vitamin D intake stratified by sun exposure related factors in the Nurses’ Health Study (NHS), and Health Professionals Follow-Up Study (HPFS)**

|  | **Quintile of vitamin D intake** | | | | | P for trend |
| --- | --- | --- | --- | --- | --- | --- |
|  | **1** | **2** | **3** | **4** | **5** |
| **Annual UV flux at residence <=** 113 | | | | | | |
| **Total vitamin D** |  |  |  |  |  |  |
| **BCC** | 1 (referent) | 1.04 ( 0.96, 1.12) | 1.11 ( 1.01, 1.21) | 1.10 ( 1.04, 1.17) | 1.10 ( 1.03, 1.17) | **<0.01** |
| **SCC** | 1 (referent) | 1.16 ( 0.84, 1.59) | 1.16 ( 0.78, 1.74) | 0.96 ( 0.60, 1.53) | 1.03 ( 0.84, 1.26) | 0.33 |
| **Melanoma** | 1 (referent) | 1.14 ( 0.90, 1.44) | 1.09 ( 0.86, 1.38) | 0.98 ( 0.77, 1.25) | 1.17 ( 0.92, 1.49) | 0.48 |
| **Dietary vitamin D** |  |  |  |  |  |  |
| **BCC** | 1 (referent) | 1.04 ( 0.98, 1.11) | 1.09 ( 1.02, 1.16) | 1.12 ( 1.05, 1.19) | 1.15 ( 1.08, 1.23) | **<0.0001** |
| **SCC** | **1 (referent)** | 1.37 ( 1.05, 1.78) | 1.06 ( 0.86, 1.31) | 1.30 ( 1.06, 1.59) | 1.27 ( 1.03, 1.56) | 0.09 |
| **Melanoma** | 1 (referent) | 1.06 ( 0.76, 1.48) | 1.09 ( 0.86, 1.39) | 1.37 ( 1.09, 1.73) | 0.93 ( 0.72, 1.19) | 1.00 |
| **Supplemental vitamin D** | **None** | **1-99 IU/d** | **100-199 IU/d** | **200-399 IU/d** | **≥ 400 IU/d** |  |
| **BCC** | 1 (referent) | 1.01 ( 0.95, 1.08) | 1.07 ( 1.00, 1.16) | 1.04 ( 0.98, 1.10) | 1.03 ( 0.97, 1.10) | 0.17 |
| **SCC** | 1 (referent) | 1.03 ( 0.85, 1.25) | 1.06 ( 0.87, 1.28) | 0.94 ( 0.78, 1.12) | 0.93 ( 0.75, 1.15) | 0.27 |
| **Melanoma** |  | 1.06 ( 0.84, 1.34) | 0.91 ( 0.72, 1.15) | 1.00 ( 0.81, 1.23) | 1.09 ( 0.85, 1.39) | 0.66 |
| **Annual UV flux at residence>** 113 | | | | | | |
| **Total vitamin D** |  |  |  |  |  |  |
| **BCC** | 1 (referent) | 1.03 ( 0.96, 1.10) | 1.06 ( 0.99, 1.15) | 1.10 ( 1.01, 1.19) | 1.10 ( 0.98, 1.24) | 0.16 |
| **SCC** | 1 (referent) | 0.96 ( 0.80, 1.16) | 1.05 ( 0.87, 1.26) | 0.79 ( 0.60, 1.03) | 1.03 ( 0.86, 1.25) | 0.72 |
| **Melanoma** | 1 (referent) | 1.12 ( 0.75, 1.66) | 1.02 ( 0.77, 1.34) | 1.12 ( 0.85, 1.48) | 0.98 ( 0.70, 1.38) | 0.77 |
| **Dietary vitamin D** |  |  |  |  |  |  |
| **BCC** | **1 (referent)** | 1.08 ( 1.01, 1.15) | 1.13 ( 1.02, 1.25) | 1.09 ( 1.02, 1.16) | 1.10 ( 1.03, 1.18) | **0.02** |
| **SCC** | **1 (referent)** | 1.12 ( 0.78, 1.61) | 1.09 ( 0.72, 1.66) | 0.96 ( 0.74, 1.25) | 1.07 ( 0.86, 1.35) | 0.94 |
| **Melanoma** |  | 1.23 ( 0.94, 1.62) | 1.27 ( 0.86, 1.87) | 1.11 ( 0.68, 1.82) | 1.25 ( 0.94, 1.66) | 0.44 |
| **Supplemental vitamin D** | **None** | **1-99 IU/d** | **100-199 IU/d** | **200-399 IU/d** | **≥ 400 IU/d** |  |
| **BCC** | 1 (referent) | 1.06 ( 0.99, 1.13) | 1.04 ( 0.96, 1.12) | 1.06 ( 0.98, 1.14) | 1.12 ( 1.00, 1.25) | 0.14 |
| **SCC** | 1 (referent) | 0.95 ( 0.72, 1.24) | 0.85 ( 0.70, 1.02) | 0.92 ( 0.71, 1.20) | 0.97 ( 0.81, 1.18) | 0.57 |
| **Melanoma** | 1 (referent) | 1.06 ( 0.82, 1.38) | 0.96 ( 0.73, 1.27) | 0.89 ( 0.70, 1.15) | 1.00 ( 0.75, 1.33) | 0.59 |
| **History of blistering sunburns=No** | | | | | | |
| **Total vitamin D** |  |  |  |  |  |  |
| **BCC** | **1 (referent)** | 1.14 ( 0.97, 1.34) | 1.20 ( 1.02, 1.41) | 1.22 ( 1.04, 1.43) | 1.34 ( 1.14, 1.57) | **<0.001** |
| **SCC** | 1 (referent) | 1.25 ( 0.77, 2.02) | 1.41 ( 0.88, 2.26) | 1.05 ( 0.51, 2.13) | 1.13 ( 0.69, 1.87) | **0.96** |
| **Melanoma** | 1 (referent) | 0.61 ( 0.31, 1.18) | 0.79 ( 0.43, 1.45) | 0.72 ( 0.31, 1.68) | 0.80 ( 0.41, 1.54) | **0.82** |
| **Dietary vitamin D** |  |  |  |  |  |  |
| **BCC** | **1 (referent)** | 1.25 ( 1.07, 1.47) | 1.16 ( 0.98, 1.36) | 1.31 ( 1.11, 1.53) | 1.36 ( 1.16, 1.60) | **<0.001** |
| **SCC** | 1 (referent) | 1.45 ( 0.78, 2.69) | 1.09 ( 0.66, 1.80) | 1.58 ( 1.00, 2.52) | 1.15 ( 0.70, 1.90) | **0.62** |
| **Melanoma** | 1 (referent) | 0.78 ( 0.41, 1.48) | 0.76 ( 0.40, 1.44) | 0.83 ( 0.40, 1.75) | 0.90 ( 0.48, 1.71) | **0.96** |
| **Supplemental vitamin D** | **None** | **1-99 IU/d** | **100-199 IU/d** | **200-399 IU/d** | **≥ 400 IU/d** |  |
| **BCC** | **1 (referent)** | 1.10 ( 0.94, 1.29) | 1.20 ( 1.03, 1.39) | 1.19 ( 1.00, 1.41) | 1.16 ( 0.99, 1.35) | **0.04** |
| **SCC** | 1 (referent) | 0.92 ( 0.31, 2.73) | 1.02 ( 0.64, 1.61) | 0.79 ( 0.51, 1.23) | 1.04 ( 0.65, 1.68) | **0.75** |
| **Melanoma** | 1 (referent) | 1.11 ( 0.57, 2.15) | 1.36 ( 0.75, 2.47) | 0.88 ( 0.41, 1.89) | 1.02 ( 0.51, 2.02) | 0.81 |
| **History of blistering sunburns=Yes** | | | | | | |
| **Total vitamin D** |  |  |  |  |  |  |
| **BCC** | 1 (referent) | 1.02 ( 0.97, 1.07) | 1.07 ( 1.02, 1.13) | 1.08 ( 1.02, 1.14) | 1.08 ( 1.02, 1.13) | 0.13 |
| **SCC** | 1 (referent) | 0.99 ( 0.86, 1.15) | 1.02 ( 0.88, 1.18) | 0.80 ( 0.69, 0.93) | 0.98 ( 0.84, 1.14) | 0.21 |
| **Melanoma** | 1 (referent) | 1.15 ( 0.86, 1.53) | 1.08 ( 0.89, 1.32) | 1.01 ( 0.80, 1.28) | 1.12 ( 0.92, 1.38) | 0.75 |
| **Dietary vitamin D** |  |  |  |  |  |  |
| **BCC** | **1 (referent)** | 1.04 ( 0.98, 1.10) | 1.10 ( 1.05, 1.16) | 1.08 ( 1.03, 1.14) | 1.10 ( 1.05, 1.16) | **<0.01** |
| **SCC** | 1 (referent) | 1.21 ( 0.89, 1.63) | 1.05 ( 0.74, 1.48) | 1.06 ( 0.84, 1.35) | 1.15 ( 0.98, 1.36) | **0.40** |
| **Melanoma** | 1 (referent) | 1.20 ( 0.99, 1.47) | 1.21 ( 0.87, 1.69) | 1.33 ( 0.90, 1.96) | 1.06 ( 0.85, 1.31) | **0.71** |
| **Supplemental vitamin D** | **None** | **1-99 IU/d** | **100-199 IU/d** | **200-399 IU/d** | **≥ 400 IU/d** |  |
| **BCC** | 1 (referent) | 1.02 ( 0.97, 1.07) | 1.02 ( 0.96, 1.10) | 1.02 ( 0.96, 1.07) | 1.07 ( 1.01, 1.12) | 0.05 |
| **SCC** | 1 (referent) | 0.93 ( 0.67, 1.28) | 0.90 ( 0.75, 1.08) | 0.91 ( 0.76, 1.08) | 0.92 ( 0.79, 1.08) | **0.18** |
| **Melanoma** | 1 (referent) | 1.02 ( 0.84, 1.25) | 0.86 ( 0.70, 1.04) | 0.90 ( 0.75, 1.07) | 1.07 ( 0.87, 1.30) | 0.99 |
| **Average time spent in direct sunlight since high school=Below median** | | | | | |  |
| **Total vitamin D** |  |  |  |  |  |  |
| **BCC** | **1 (referent)** | 1.05 ( 0.96, 1.14) | 1.11 ( 1.02, 1.20) | 1.15 ( 1.06, 1.25) | 1.19 ( 1.10, 1.29) | **<0.01** |
| **SCC** | **1 (referent)** | 1.03 ( 0.82, 1.30) | 1.12 ( 0.90, 1.40) | 0.84 ( 0.67, 1.06) | 0.92 ( 0.73, 1.16) | 0.11 |
| **Melanoma** | 1 (referent) | 0.84 ( 0.53, 1.33) | 0.92 ( 0.67, 1.27) | 0.94 ( 0.68, 1.29) | 1.19 ( 0.87, 1.62) | 0.09 |
| **Dietary vitamin D** |  |  |  |  |  |  |
| **BCC** | **1 (referent)** | 1.08 ( 1.00, 1.17) | 1.14 ( 1.01, 1.27) | 1.13 ( 1.05, 1.22) | 1.13 ( 1.04, 1.23) | **<0.01** |
| **SCC** | **1 (referent)** | 1.13 ( 0.65, 1.97) | 1.07 ( 0.86, 1.34) | 0.97 ( 0.64, 1.48) | 1.05 ( 0.83, 1.32) | 0.72 |
| **Melanoma** | 1 (referent) | 1.13 ( 0.82, 1.56) | 1.23 ( 0.90, 1.68) | 1.29 ( 0.83, 1.99) | 0.94 ( 0.66, 1.32) | 0.81 |
| **Supplemental vitamin D** | **None** | **1-99 IU/d** | **100-199 IU/d** | **200-399 IU/d** | **≥ 400 IU/d** |  |
| **BCC** | **1 (referent)** | 1.08 ( 0.93, 1.25) | 1.08 ( 0.94, 1.24) | 1.09 ( 1.02, 1.17) | 1.15 ( 1.06, 1.24) | **<0.001** |
| **SCC** | **1 (referent)** | 0.97 ( 0.47, 2.01) | 0.89 ( 0.61, 1.31) | 0.94 ( 0.65, 1.37) | 0.85 ( 0.66, 1.09) | 0.11 |
| **Melanoma** | 1 (referent) | 0.90 ( 0.65, 1.25) | 0.93 ( 0.68, 1.28) | 1.00 ( 0.58, 1.71) | 1.34 ( 0.94, 1.91) | 0.29 |
| **Average time spent in direct sunlight since high school=Above median** | | | | | |  |
| **Total vitamin D** |  |  |  |  |  |  |
| **BCC** | 1 (referent) | 1.04 ( 0.96, 1.12) | 1.10 ( 1.02, 1.18) | 1.09 ( 0.98, 1.21) | 1.08 ( 1.00, 1.16) | 0.20 |
| **SCC** | 1 (referent) | 1.07 ( 0.87, 1.31) | 1.12 ( 0.79, 1.57) | 0.87 ( 0.69, 1.09) | 1.10 ( 0.88, 1.37) | 0.97 |
| **Melanoma** | 1 (referent) | 1.19 ( 0.64, 2.20) | 1.07 ( 0.65, 1.78) | 1.06 ( 0.71, 1.58) | 1.00 ( 0.60, 1.69) | 0.57 |
| **Dietary vitamin D** |  |  |  |  |  |  |
| **BCC** | **1 (referent)** | 1.00 ( 0.93, 1.08) | 1.07 ( 1.00, 1.15) | 1.07 ( 1.00, 1.15) | 1.13 ( 1.05, 1.21) | 0.08 |
| **SCC** | **1 (referent)** | 1.39 ( 1.13, 1.71) | 1.12 ( 0.76, 1.65) | 1.25 ( 1.01, 1.56) | 1.44 ( 1.15, 1.79) | 0.01 |
| **Melanoma** | **1 (referent)** | 1.18 ( 0.90, 1.55) | 1.07 ( 0.67, 1.73) | 1.08 ( 0.61, 1.93) | 1.18 ( 0.88, 1.58) | 0.62 |
| **Supplemental vitamin D** | **None** | **1-99 IU/d** | **100-199 IU/d** | **200-399 IU/d** | **≥ 400 IU/d** |  |
| **BCC** | 1 (referent) | 1.01 ( 0.89, 1.16) | 1.05 ( 0.90, 1.22) | 0.99 ( 0.92, 1.05) | 1.03 ( 0.96, 1.11) | 0.57 |
| **SCC** | 1 (referent) | 0.85 ( 0.69, 1.05) | 0.87 ( 0.71, 1.07) | 0.78 ( 0.64, 0.95) | 0.92 ( 0.73, 1.16) | 0.27 |
| **Melanoma** | 1 (referent) | 1.13 ( 0.86, 1.47) | 0.95 ( 0.72, 1.25) | 0.93 ( 0.72, 1.21) | 0.91 ( 0.66, 1.26) | 0.57 |
| ^*^ Adjusted for family history of melanoma, natural hair color, number of arm moles, sunburn susceptibility as a child/adolescent, number of lifetime blistering sunburns, average time spent in direct sunlight since high school, cumulative UV flux since baseline**,** body mass index, physical activity, smoking status, intakes of total energy, alcohol, coffee and citrus intake. Among women analyses were additionally adjusted for menopausal status and postmenopausal hormone use. | | | | | | |
