# Supplementary material for: Vitamin D Intake and Risk of Skin Cancer in US Women and Men
Source: PLoS One. 2016 Aug 24;11(8):e0160308. doi: 10.1371/journal.pone.0160308 (PMC4996491; doi:10.1371/journal.pone.0160308)
Supplement: S2 Table — (DOC) [file pone.0160308.s002.doc]

**Supplemental Table 2. Pooled hazard ratios^*^ (and 95% confidence intervals) of Squamous Cell Carcinoma and Melanoma by intake of vitamin D rich food in the Nurses’ Health Study (NHS), and Health Professionals Follow-Up Study (HPFS)**

|  | **Quintile of vitamin D rich food intake** | | | | | P for trend |
| --- | --- | --- | --- | --- | --- | --- |
|  | **1** | **2** | **3** | **4** | **5** |
| **Total Fish** |  |  |  |  |  |  |
| **SCC** | 1 (referent) | 1.07 ( 0.94, 1.22) | 1.01 ( 0.89, 1.16) | 0.97 ( 0.85, 1.11) | 0.93 ( 0.74, 1.17) | 0.22 |
| **Melanoma** | 1 (referent) | 1.02 ( 0.85, 1.22) | 0.95 ( 0.67, 1.36) | 1.10 ( 0.92, 1.32) | 1.17 ( 0.98, 1.41) | 0.12 |
| **White Fish** |  |  |  |  |  |  |
| **SCC** | 1 (referent) | 1.04 ( 0.92, 1.19) | 0.97 ( 0.84, 1.12) | 1.02 ( 0.88, 1.17) | 0.99 ( 0.86, 1.14) | 0.80 |
| **Melanoma** | 1 (referent) | 1.25 ( 1.04, 1.50) | 1.14 ( 0.94, 1.39) | 1.21 ( 1.00, 1.46) | 1.27 ( 1.05, 1.54) | 0.07 |
| **Tuna** |  |  |  |  |  |  |
| **SCC** | 1 (referent) | 1.00 ( 0.82, 1.21) | 0.94 ( 0.82, 1.08) | 0.97 ( 0.85, 1.11) | 0.94 ( 0.75, 1.18) | *0.38* |
| **Melanoma** | 1 (referent) | 1.05 ( 0.88, 1.25) | 1.10 ( 0.92, 1.33) | 1.03 ( 0.86, 1.23) | 1.11 ( 0.93, 1.33) | *0.53* |
| **Total Dairy Food** |  |  |  |  |  |  |
| **SCC** | 1 (referent) | 1.19 ( 1.01, 1.42) | 1.19 ( 1.03, 1.37) | 1.06 ( 0.91, 1.23) | 1.25 ( 1.07, 1.47) | 0.10 |
| **Melanoma** | 1 (referent) | 1.10 ( 0.79, 1.53) | 1.19 ( 0.91, 1.56) | 1.10 ( 0.91, 1.34) | 1.01 ( 0.82, 1.26) | 0.78 |
| **Total Milk** |  |  |  |  |  |  |
| **SCC** | 1 (referent) | 1.10 ( 0.95, 1.26) | 1.18 ( 1.03, 1.35) | 1 1.15 ( 1.00, 1.33) | 1.11 ( 0.96, 1.28) | 0.16 |
| **Melanoma** | 1 (referent) | 1.15 ( 0.96, 1.38) | 1.05 ( 0.88, 1.26) | 1.07 ( 0.89, 1.29) | 0.97 ( 0.80, 1.18) | 0.49 |
| **Skim or low fat Milk** |  |  |  |  |  |  |
| **SCC** | 1 (referent) | 1.07 ( 0.90, 1.27) | 1.18 ( 0.97, 1.43) | 1.12 ( 0.97, 1.29) | 1.10 ( 0.90, 1.35) | 0.19 |
| **Melanoma** | 1 (referent) | 1.02 ( 0.85, 1.23) | 1.06 ( 0.88, 1.27) | 1.06 ( 0.88, 1.27) | 0.96 ( 0.79, 1.17) | 0.78 |
| **Breakfast Cereal** |  |  |  |  |  |  |
| **SCC** | 1 (referent) | 0.84 ( 0.73, 0.97) | 0.93 ( 0.82, 1.07) | 0.90 ( 0.79, 1.04) | 0.95 ( 0.83, 1.09) | 0.96 |
| **Melanoma** | 1 (referent) | 1.16 ( 0.87, 1.55) | 1.26 ( 1.00, 1.60) | 1.25 ( 0.86, 1.81) | 1.19 ( 0.98, 1.45) | 0.10 |
| **Egg** |  |  |  |  |  |  |
| **SCC** | 1 (referent) | 0.93 ( 0.82, 1.06) | 0.90 ( 0.80, 1.03) | 0.89 ( 0.76, 1.05) | 0.89 ( 0.76, 1.04) | 0.16 |
| **Melanoma** | 1 (referent) | 1.19 ( 1.00, 1.42) | 1.04 ( 0.87, 1.25) | 1.17 ( 0.98, 1.39)) | 1.14 ( 0.92, 1.41) | 0.26 |
| ^*^ Adjusted for family history of melanoma, natural hair color, number of arm moles, sunburn susceptibility as a child/adolescent, number of lifetime blistering sunburns, average time spent in direct sunlight since high school, cumulative UV flux since baseline**,** body mass index, physical activity, smoking status, intakes of total energy, alcohol, coffee and citrus intake. Among women analyses were additionally adjusted for menopausal status and postmenopausal hormone use. | | | | | | |
